# Functional Analysis of CRISPR-Cas9-Mediated Gene Deletion in *E. coli* DH5α on Membrane Permeability and Transformation Efficiency

**DOI:** 10.3390/microorganisms14010198

**Published:** 2026-01-15

**Authors:** Feifan Leng, Xinyi Liu, Jinli He, Yubo Wang, Ning Zhu, Xiaopeng Guo, Wen Luo, Yonggang Wang

**Affiliations:** 1 School of Life Science and Engineering, Lanzhou University of Technology, Pengjiaping Road 36, Qilihe District, Lanzhou 730050, China; 2 School of Petrochemical Engineering, Lanzhou University of Technology, Lanzhou 730050, China

**Keywords:** CRISPR/Cas9, the kinetic model, membrane permeability, transformation mechanism, cell structure

## Abstract

This research utilized the CRISPR/Cas9 editing method to generate six mutant strains of *Escherichia coli *(*E. coli*) DH5α targeting specific genes. The functional characterization and phenotypic analysis confirmed the regulatory roles of these genes in modifying membrane permeability. The variations in membrane permeability among the mutant strains were assessed by measuring electrical conductivity, ortho-nitrophenyl-β-D-galactopyranoside (ONPG) hydrolysis, and propidium iodide (PI) fluorescence, with *E. coli* DH5α:*ompA′* exhibiting the most pronounced increase in membrane permeability. The function of these genes in transformation was analyzed from physicochemical and microscopic perspectives. Assays of plasmid transformation efficiency revealed a significant enhancement in the *E. coli* DH5α:*ompA′* mutant strain, underscoring the critical function of outer membrane proteins in DNA acquisition. Permeability simulations were performed utilizing the *E. coli* DH5α:*ompA′* mutant strain, grounded in a previously established model. The quantitative correlation between transformation efficiency and membrane permeability in this mutant conformed to the equation T = aP + c.

## 1. Introduction

The cell membrane consists of the inner membrane (IM), the outer membrane (OM), and the peptidoglycan layer [1], which are essential for sustaining cellular homeostasis and facilitating interactions between the cell and its external environment. The OM serves as both a physical barrier to external stress and toxins and a vital interface that governs the transmembrane movement of nutrients, signaling chemicals, and genetic material [2,3]. Outer membrane-associated proteins, channel proteins, and regulatory factors are crucial in influencing membrane permeability and cellular adaptation to external conditions [4].  

Bacterial genetic transformation is a primary mechanism of horizontal gene transfer (HGT), allowing bacteria to assimilate exogenous DNA under particular environmental circumstances and incorporate it into their genome [5]. This method enables bacteria to swiftly obtain new genetic characteristics and improve their resilience to environmental fluctuations [6]. Contemporary investigations into the mechanisms of natural transformation in bacteria have predominantly concentrated on species exhibiting natural competence [7], such as *Bacillus subtilis*, *Haemophilus influenzae*, and *Neisseria gonorrhoeae* [8]. These bacteria have been shown to exhibit specific protein complexes that facilitate DNA binding and transmembrane transport [9]. Nevertheless, in contrast to these species, the mechanism of DNA uptake in *Escherichia coli* (*E. coli*) is inadequately comprehended and needs thorough characterization. Traditionally, *E. coli* is not regarded as a bacterium that undergoes spontaneous plasmid transformation [10], with transformation predominantly reliant on artificially induced techniques, including pretreatment with high concentrations of divalent metal ions (such as CaCl_2_), followed by subsequent steps such as heat shock, polyethylene glycol treatment, or ultrasonic treatment [11,12,13]. Recent investigations indicate that under specific natural settings, *E. coli* can assimilate exogenous DNA, such as from ambient water or food extracts or during repeated freeze–thaw cycles [14,15]. These data indicate that the transformation capability of *E. coli* is not just influenced by external physical or chemical stimuli; endogenous genetic variables also significantly contribute to this process.

Prior research has demonstrated that genes responsible for OM proteins, periplasmic elements, and global stress regulatory factors can influence membrane permeability and transformation efficiency by altering membrane architecture or cellular stress conditions [16]. Wan et al. utilized transcriptome and proteomic methodologies, revealing an increase in the *loiP* gene in response to CaCl_2_ therapy [17]. Subsequent evaluations of cellular morphology, transformation efficiency, and membrane properties indicated no substantial discrepancies between the Δ*loiP* mutant strain and the wild-type *E. coli* DH5α strain regarding morphology, growth kinetics, or IM permeability. The transformation efficacy of plasmids of differing sizes was significantly diminished in the mutant strain, and OM permeability dropped by a factor of 2.94. The findings demonstrate that the *loiP* gene is essential for regulating transformation efficiency and preserving OM integrity in the transformation capability of *E. coli*. Zhu et al. [18]. incorporated transcriptomics, proteomics, and correlation analysis to uncover essential genes implicated in the development of Ca^2+^-mediated transformation capabilities in *E. coli* DH5α. Employing red recombination technology, they produced three single-gene knockout strains (Δ*yiaW*, Δ*ygiZ*, and Δ*osmB*). The deletion of *yiaW*, *ygiZ*, or *osmB* markedly improved the transformation efficiency for plasmids of differing sizes. Modifications in membrane architecture may enhance DNA ingress by diminishing the barrier efficacy of the OM, whereas stress regulatory networks may affect DNA recognition, binding, transmembrane transport, or intracellular processing [9,19]. Nonetheless, the precise functions of different genes in these processes remain ambiguous, and it is now uncertain whether alterations in membrane permeability alone can entirely explain the observed variations in transformation efficiency. In our prior study, we developed a quantitative correlation model elucidating the relationship between transformation efficiency and membrane permeability in *E. coli*, based on measurements of membrane permeability and assessments of transformation efficiency [20]. A transcriptional study discovered a set of genes linked to alterations in membrane permeability. This work offered significant insights into the correlation between membrane characteristics and transformation efficacy. Nevertheless, the model was fundamentally a correlation study and failed to include direct genetic perturbations, rendering it hard to ascertain definitive conclusions regarding the causative roles of individual genes in the regulation of membrane permeability and the process of DNA uptake.  

The current study utilized CRISPR-Cas9 gene editing technology to create six gene deletion mutants (*ybaV′*, *rpoS′*, *ycaI* *ompA′*, *ompR′*, and *hofC′*) in *E. coli* DH5α. The impact of these gene deletions on membrane permeability, cellular stress response, and plasmid transformation efficiency was methodically examined. These genes are involved in preserving the OM structure, global transcriptional regulation, fimbriae assembly, and possible DNA absorption pathways, each demonstrating unique functional specialization. This study combined the gene knockout strategy with quantitative analysis of membrane properties and transformation assays to introduce gene-level causal validation based on an established correlation model. This study enhances the comprehension of the regulatory processes governing *E. coli* transformation and offers a theoretical foundation for the systematic improvement of transformation procedures and the creation of highly efficient modified strains.

## 2. Materials and Methods

### 2.1. Plasmid and Culture Conditions

*E. coli* DH5α and plasmid pUC19 were supplied by the Microbial Genetic Engineering Laboratory, School of Life Science and Engineering, Lanzhou University of Technology, Lanzhou, China. The plasmid was introduced into the *E. coli* DH5α recipient strain, which was grown in Lysogeny Broth (LB) medium at 37 °C. The plasmid pUC19 harbors the ampicillin resistance (Amp^r^) gene, facilitating the selection of cells possessing the pUC19 plasmid through a selection medium containing 100 μg/mL of ampicillin. The plasmid pCas9 (#171139; https://www.addgene.org/171139/ (accessed on 10 January 2026)), which can express the Cas9 protein, tracrRNA, and gRNA in *E. coli*, was acquired from Addgene.

### 2.2. Confirmation and Construction of Mutant Strains

#### 2.2.1. Screening of Membrane-Related Genes

Based on previous research and preliminary work in our laboratory, we identified six genes associated with the OM of *E. coli*. These genes include the OM protein *ompA* [21]; the DNA-binding transcriptional regulator *ompR* [22], which modulates the expression of OM porins such as *ompC* and *ompF* [23]; and the stress-related sigma factor *rpoS* [24], which plays a crucial role under conditions of oxidative stress, high osmotic pressure, and other stress factors [25]. Additionally, we identified the membrane protein *ycaI* [26], which belongs to the metal-β-lactamase superfamily, and the *hofC* gene, which encodes a protein homologous to *pilC* from Pseudomonas aeruginosa [27]. Furthermore, the *ybaV* gene encodes a protein that is regulated by Crp and Sxy through a CRP-S promoter [28]. Potential interactions among the encoded proteins were investigated using the STRING database (https://string-db.org (accessed on 10 January 2026)), leading to the construction of a protein–protein interaction network diagram [29].

#### 2.2.2. sgRNA Assembly for Mutant Strains

The single guide RNA (sgRNA) was developed utilizing the CRISPR design tool CHOPCHOP-V3 (http://chopchop.cbu.uib.no (accessed on 10 January 2026)), and its targeting specificity was confirmed through Sanger sequencing [30]. A 20-base-pair spacer sequence corresponding to the target gene was identified for acquisition. To enhance the ligation efficiency between the pCas9 plasmid and the newly introduced spacer sequence, a linker sequence was designed at the 5’ end of the spacer. This process involved designing a pair of primers: the upstream primer containing the “AAAC” adapter sequence and the downstream primer containing the “AAAAC” adapter sequence. After annealing, this pair of primers formed a double-stranded oligonucleotide. The designed primers, which were synthesized with PAGE purity, were commissioned from Shanghai Shenggong Bioengineering Co., Ltd., Shanghai, China. The sequences of the synthetic primers were detailed in Table S1.

#### 2.2.3. Oligo Annealing and Plasmid Construction

Following the synthesis process, the two single-stranded oligonucleotides were diluted to a concentration of 10 μmol/L and subjected to a denaturation-annealing procedure to facilitate the formation of dsDNA, in accordance with the reaction parameters outlined in Table S2. The pCas9 plasmid was linearized by digestion with the *BsaI* restriction endonuclease, which targets specific recognition sites located between adjacent repeat elements, and the resulting overhangs match the vector-specified adapters on the annealed, 5′-phosphorylated spacer oligos (the digestion protocol is detailed in Table S3), thereby creating a gene-specific CRISPR/Cas9 system designed to target *E. coli* cells (the ligation conditions are specified in Table S3). The reaction mixture was gently mixed using a micropipette or briefly vortexed, followed by centrifugation at room temperature to consolidate the solution. Ligation was performed at 16 °C for 16 h, after which the resultant product was transformed into the competent cells [31].

#### 2.2.4. Validation of sgRNA Cloning via Colony PCR

Uniform white colonies were selected and resuspended in 10 μL of sterile double-distilled water (ddH_2_O) within PCR tubes. A 2 μL aliquot of the bacterial suspension was used as a template for colony PCR to amplify the DNA encompassing the sgRNA insertion site to confirm the directed cloning of the sgRNA into the pCas9 plasmid. The primer sequences and PCR settings employed for specificity validation are detailed in Table S4. Following the analysis of PCR results using agarose gel electrophoresis, samples exhibiting bands of the anticipated size were classified as positive clones [32].

#### 2.2.5. Validation of Gene Knockout Through Unidirectional Sequencing Analysis

Gene knockout efficacy was verified through unidirectional Sanger sequencing using specific upstream primers, with detailed primer sequences and reaction conditions provided in Table S4. Target gene fragments were amplified with specifically designed primers and separated by agarose gel electrophoresis. Purified PCR products (generated using high-fidelity DNA polymerase such as PrimeSTAR^®^ Max) were submitted to Sangon Biotech (Shanghai) Co., Ltd., Shanghai, China, for unidirectional Sanger sequencing. The continuity of sequences flanking the deletion sites was analyzed to confirm successful gene knockout. Wild-type strain controls were included throughout the experimental process, and all experiments were conducted in three independent replicates to ensure reproducibility. All raw sequencing data and analytical results have been archived for verification purposes.  

### 2.3. Validation of Gene Knockout Through Quantitative PCR

Gene knockout was achieved by disrupting the coding sequence or regulatory regions of target genes, leading to significant reduction or complete absence of mRNA expression [33]. The mRNA levels of the target gene in the knockout mutants and wild-type controls were quantitatively compared through quantitative PCR (qPCR) to further validate whether the gene knockout was successful. Primers for real-time qPCR (listed in Table S5) were designed according to standard principles and verified for specificity using BLAST (version 2.16.0) (https://blast.ncbi.nlm.nih.gov/Blast.cgi (accessed on 10 January 2026)). Total RNA was extracted from mutant and control strains using the BioTeke High-Purity RNA Rapid Extraction Kit (BioTeke Corporation, Beijing, China). Reverse transcription was performed with the Honor™ II 1st Strand cDNA Synthesis SuperMix (HonorGene Biotechnology, Changsha, China) for qPCR (gDNA digester plus), ensuring equivalent RNA input across samples. Quantitative PCR was conducted using the Unique Aptamer™ qPCR SYBR^®^ Green Master Mix (Low Rox Plus) (Unique Biotechnology, Shanghai, China) under the following thermal cycling conditions: 95 °C for 5 min, followed by 40 cycles of 95 °C for 10 s and 60 °C for 30 s. Relative gene expression was calculated using the 2^−ΔΔCt^ method, with 16S rRNA serving as the endogenous reference. All experiments were independently replicated at least three times to ensure statistical robustness [17].

### 2.4. Measurement of Growth Curves for Mutant Strains

The growth curves of the wild-type strain *E. coli* DH5α and the six mutant strains (*rpoS′*, *ompA′*, *ompR′*, *ycaI′*, *hofC′*, *ybaV′*) were determined using a micro-plate reader. Each strain was initially cultured overnight in LB medium at 37 °C, with LB medium as a blank control and the wild-type strain as a positive control. Following this initial culture, the strains were inoculated into 50 mL of LB liquid medium, achieving an initial optical density OD_600_ of 0.05 ± 0.002, at a dilution ratio of 1:100. All cultures were maintained at 37 °C with shaking at 180 rpm, and each strain underwent three biological replicates. The growth of the strains was continuously monitored until they reached the stationary phase, with the entire experimental process lasting approximate 24 h. A growth curve was subsequently constructed, using time as the independent variable and the OD_600_ values as the dependent variable [34].  

### 2.5. Measurement of Transformation Efficiency in Mutant Strains

To systematically assess the influence of membrane-related genes on DNA transformation under various external stimuli, this study designed three transformation systems: chemical transformation, ultrasound-mediated transformation, and natural transformation. These systems correspond to distinct mechanisms for regulating membrane permeability, with differences in key parameters detailed in Table S6.

#### 2.5.1. Chemical Transformation Efficiency

This system aims to examine the regulatory function of genes in Ca^2+^-induced membrane permeability. Each strain was subcultured at a 5% inoculation rate into fresh LB liquid media and incubated at 37 °C with shaking at 180 rpm until the optical density at 600 nm reached approximately 0.5 ± 0.02. The culture was subsequently frozen on ice for 10 min, after which 1.5 mL of the bacterial solution was centrifuged at 6500 rpm for 5 min. The supernatant was removed, and the pellet was resuspended in 0.75 mL of ice-cold 100 mM CaCl_2_ solution, then incubated on ice for 30 min. Subsequent to an additional centrifugation stage, the bacterial cells were resuspended in 0.1 mL of pre-chilled CaCl_2_ solution, combined with 100 ng of pUC19 plasmid, and maintained on ice for 30 to 45 min. The combination was thereafter subjected to heat shock at 42 °C for 90 s and promptly transferred to an ice-water bath for 5 min. Subsequently, 900 μL of pre-warmed SOC media was introduced, and the cells were left to recuperate at 37 °C while agitating them at 200 rpm for 1 h. The modified bacterial suspension was serially diluted, applied to LB plates with 100 μg/mL ampicillin, and incubated at 37 °C for 16–18 h prior to enumerating the transformants. As a negative control, a bacterial slurry devoid of plasmid was inoculated onto LB plates lacking antibiotics to eliminate the impact of spontaneous resistance mutations.

#### 2.5.2. Establishment of Ultrasound-Mediated Plasmid Transformation System

**Figure 1:**
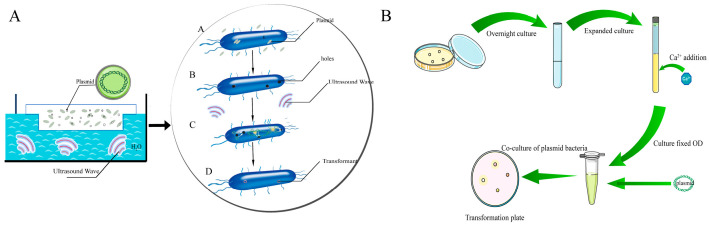
DNA transfer process. (**A**) The process of ultrasound-induced DNA transfer. (**B**) Establishment of plasmid transformation system.

This study built an ultrasound-mediated transformation system to test the effect of physical stimulation on membrane permeability and to assess the unique role of pertinent membrane genes under these conditions. *E. coli* DH5α was cultivated in 50 mL of LB liquid medium until it attained the logarithmic growth phase, exhibiting an OD_600_ of 0.5 to 0.6. The cell pellet was subsequently collected, rinsed twice with physiological saline (0.85% NaCl), and resuspended in 0.75 mL of ice-cold CaCl_2_ solution (0.1 M). Plasmid pUC19 (final concentration 1 ng/μL) was introduced to the cell suspension, which was thereafter subjected to sonication (40 kHz, 300 W, 12 s). Thereafter, LB liquid medium was incorporated, and the combination was incubated at 37 °C for one hour. A volume of 100 μL of the mixture was applied to LB-Amp (100 μg/mL) agar plates and incubated overnight at 37 °C for transformation assessment. Three replicates were conducted for each group, and the transformation efficiency was determined by calculating the number of transformants per 1 μg of transforming DNA. Transformation Efficiency = (Number of Transformants × Dilution Factor)/Plasmid DNA Utilized (μg) × 100%. The plasmid concentration was standardized across all transformation systems. The bacterial DNA transformation process was illustrated using ultrasound (Figure 1A).

#### 2.5.3. Plasmid Incubation-Based Transformation Condition

By creating an artificial transformation system that mimics natural conditions, we can enhance our comprehension of the physiological mechanisms by which bacteria assimilate DNA through the basal membrane in the absence of significant external stimulation. *E. coli* was cultivated overnight in LB medium, followed by the addition of CaCl_2_ to attain a final concentration of 100 mM, until it reached the logarithmic growth phase. 1 μg of plasmid pUC19 was inserted and incubated at 37 °C with shaking at 180 rpm for approximately 1 h. Two hundred microliters of the culture were extracted and disseminated onto an LB selection plate with 100 μg/mL ampicillin, then incubated at 37 °C. Each experimental group was executed in triplicate (Figure 1B).

### 2.6. Investigation of the Properties of Cell Membranes in Mutant Strains

#### 2.6.1. Morphological Measurement of Mutant Strains by SEM and AFM

Six mutant strains of *E. coli*, along with their wild-type counterparts, were inoculated into LB liquid medium and cultured at 37 °C and 200 rpm until they reached the logarithmic growth phase. The *E. coli* DH5α suspension at the logarithmic growth phase was centrifuged at 3000 rpm for 5 min to collect the cells, while the supernatant was discarded. The cell pellet was washed twice with PBS buffer and resuspended in PBS to remove any residual components of the culture medium that might interfere with the subsequent fixation process. The cell pellet was then fixed with 2.5% glutaraldehyde for 2 h. Glutaraldehyde fixation crosslinks cellular proteins, preserving cell structure and ensuring that the morphology of the cells remains unchanged during subsequent processing. The cell pellet was washed three times with sterile water to remove surface impurities, followed by sequential washes with ethanol gradients (30%, 50%, 70%, 80%, 90%, 100%) and tert-butanol. This gradual dehydration process helps prevent cell distortion due to abrupt changes in water content. The samples were then resuspended in tert-butanol, freeze-dried, and observed using scanning electron microscopy (SEM) (JEOL Ltd., Tokyo, Japan).

To further evaluate the effects of ultrasound on bacterial cells, high-resolution imaging and mechanical measurements were performed using atomic force microscopy (AFM) (Agilent Technologies, Santa Clara, CA, USA). After fixation with 2.5% glutaraldehyde for 15 min, 20 μL of the bacterial suspension was placed on a natural mica substrate, air-dried, and analyzed using AFM in tapping mode. The scanning area was 10 μm × 10 μm, and AFM was used to capture morphology, phase, and 3D images to qualitatively examine cell surface characteristics. The images were processed using Gwyddion software (version 3.1) without further modifications.

#### 2.6.2. Electrical Conductivity of Mutant Strains

The strain treatment procedure adhered to the protocol specified in Section 2.4. In addition, six mutant strains of *E*. *coli* were subjected to ultrasonic treatment at a power of 300 W for 12 s. Following this, the bacterial liquid was processed into a bacterial suspension employing the previously mentioned two methods. Both the standard bacterial suspension and the sonicated bacterial suspension were subsequently introduced into a conductivity measurement cell, where the variations in the conductivity of the solution were continuously monitored in real time utilizing a high-precision conductivity meter [35].

#### 2.6.3. Detection of Altered Membrane Permeability Using Propidium Iodide (PI)

PI is a nucleic acid stain that is unable to penetrate an intact cell membrane. However, upon damage to the cell membrane, PI can enter the cell and bind to DNA, resulting in the emission of red fluorescence. Consequently, the fluorescence intensity serves as a direct indicator of the extent of cell membrane integrity disruption; increased membrane permeability allows for greater PI uptake, leading to elevated fluorescence readings [36]. Following the protocol outlined in Section 2.5.1 for processing the bacterial suspension, PI was added to achieve a final concentration of 50 μg/mL. The mixture was then incubated at 37 °C in the dark for 30 min before measurement using a fluorescence spectrophotometer (Shanghai Lengguang Technology Co., Ltd., Shanghai, China). Wild-type *E. coli* DH5α will serve as the control group, with three replicates established for each experimental condition.

#### 2.6.4. Measurement of Intracellular Membrane Permeability of Mutant Strains

β-galactosidase is an inducible endoenzyme found in *E*. *coli*, which is progressively released from the cytoplasm following the disruption of the bacterial IM. The hydrolysis of Ortho-nitrophenyl-β-D-galactopyranoside (ONPG) hydrolysis by β-galactosidase results in the formation of the yellow compound Ortho-nitrophenyl (ONP), which exhibits absorbance at a wavelength of 420 nm [37]. The activity of β-galactosidase can thus be quantified by measuring the absorbance at this specific wavelength. Consequently, alterations in intimal permeability were evaluated by monitoring the production of ONP under various sonication treatments. In accordance with Section 2.5.1, a sample consisting of 190 µL of *E. coli* suspension and 10 µL of a 30 mM ONPG solution (totaling 200 µL) was thoroughly mixed and incubated at 37 °C for 30 min, after which the absorbance at 420 nm for each mixed solution was recorded.

### 2.7. Variations in Membrane Permeability and the Establishment of a Kinetic Model of E. coli DH5α Under Different Ultrasonic Conditions

#### 2.7.1. Measurement of Cell Membrane Permeability

Fluorescein diacetate (FDA) staining was used for measurement. *E. coli* DH5α cells in the logarithmic phase were resuspended in ice-cold CaCl_2_ solution and subjected to ultrasonic treatment at different power levels. After treatment, FDA stock solution was added to achieve a final concentration of 0.25 mg/mL, and the mixture was incubated in the dark at 20 °C for 5 min. Fluorescence intensity was then measured using a fluorescence spectrophotometer (excitation wavelength 269 nm, emission wavelength 517 nm). Three biological replicates were set for each power condition.

#### 2.7.2. Simulation of the Kinetic Model of *E. coli* Cell Membrane Permeability

Based on experimental conditions and mass transfer theory, the following assumptions were made for the model: (1) The rate of permeable substance loss due to cell death is constant, denoted as k_1_; (2) The inward mass transfer rate N_2_ is proportional to the extracellular concentration F (with coefficient k_2_), and the outward mass transfer rate N_3_ is proportional to the intracellular concentration E (with coefficient k_3_); (3) The total amount of permeable substances inside and outside the cell is T, satisfying F = T − E; (4) Bidirectional transport occurs simultaneously. Based on these assumptions, we derived a kinetic equation to describe the change in intracellular permeable substance concentration over time. To apply the model to experimental data, we used nonlinear regression analysis to fit the above equation with the FDA fluorescence intensity-time data measured under different ultrasound power levels, in order to obtain the best model parameters. The goodness of fit of the model was assessed by calculating the coefficient of determination (R^2^).

### 2.8. Analysis of Fourier Transform Infrared Spectroscopy (FT-IR)

To verify the structural alterations in various mutant strains, the treatment protocol for *E. coli* was consistent with that described in Section 2.6.2. Subsequently, the bacteria were rinsed and resuspended in sterile PBS buffer to obtain a bacterial suspension. The bacterial cell samples were then vacuum freeze-dried, followed by freeze-drying and stored in a desiccator to eliminate all moisture. A small amount of the dried sample was taken, thoroughly ground with KBr powder at a ratio of 50–100 times the sample mass, and pressed into pellets using a pellet press. KBr pellets were prepared separately as blank controls. Fourier-transform infrared spectroscopy (Nexus670 FT-IR, Nicolet, Madison, WI, USA) was used to scan and analyze the samples in the wavelength range of 4000–600 cm^−1^. An average spectrum was obtained for each sample from three parallel replicates, followed by baseline correction processing [38].

### 2.9. Statistical Analysis

All experiments were conducted with three replicates, and the data were presented as the mean ± standard deviation. Experimental data were input into SPSS 20.0 statistical software for one-way analysis of variance and Tukey’s test to determine significant differences. A significance level of *p* < 0.05 indicated the presence of significant differences, while *p* > 0.05 indicated no significant differences.

## 3. Results

### 3.1. Schematic of Interactions Within a Gene Network

To explore regulatory genes potentially implicated in *E. coli* transformation, six genes encoding proteins with varied cellular roles were analyzed (Table S7). *OmpA*, *ompR*, and *rpoS* are linked to membrane integrity and the control of stress responses, whereas *ycaI*, *hofC*, and *ybaV* have been identified as membrane-associated genes exhibiting homology to competence-related proteins in other bacterial species. Sequence alignment identified conserved regions throughout these genes, suggesting possible functions in DNA absorption and membrane-related activities. Furthermore, STRING network and chromosomal localization analyses indicated that these genes constitute a functional interaction network in *E. coli* (Figure S2A), dispersed across various genomic locations, suggesting structural diversity and specific cellular roles (Figure S2B). Collectively, these data corroborate their role in membrane integrity, stress response, and competence development.

### 3.2. Gene Knockout of Target Genes

#### 3.2.1. Construction of CRISPR-Cas9 Plasmids Containing sgRNAs of Variable Lengths

**Figure 2:**
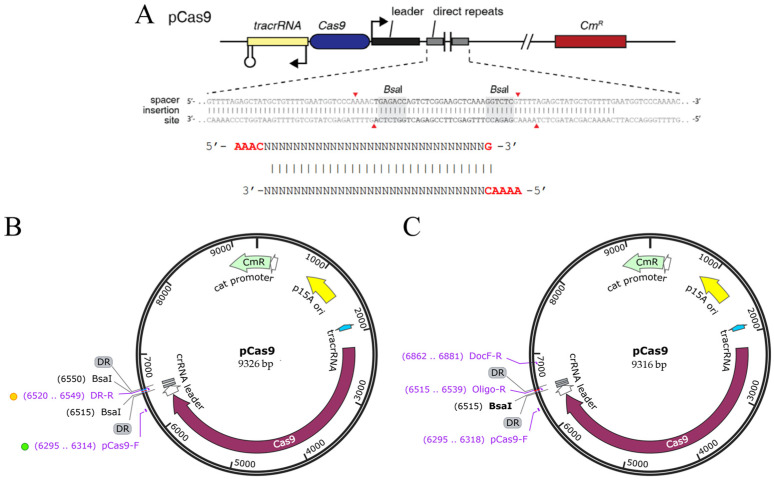
pCas9 vector and constructed vectors. (**A**) Insertion site map of sgRNA into the pCas9 plasmid, The red triangles indicate the spacer insertion sites; (**B**) pCas9 vector; (**C**) Schematic diagram of the constructed sgRNA-pCas9 plasmid.

Utilizing the conserved Protospacer Adjacent Motif (PAM) characteristics of the CRISPR-Cas9 system, target sites for sgRNA were predicted through the use of the ChopChop online tool (version 3). Given the compactness of prokaryotic genomes, the generally low off-target propensity of CRISPR systems in bacteria, and the lack of standardized sgRNA-design algorithms for *E. coli*, the two top-ranked candidate sequences based on ChopChop scores were selected, with their insertion sites illustrated in Figure 2A [39]. Each sgRNA consists of a 20 bp target-specific sequence upstream of the PAM, with nucleotide compositions detailed as follows. The plasmid maps before and after sgRNA insertion were shown in Figure 2B,C.

#### 3.2.2. Verification of Plasmid pCas9 Through Restriction Enzyme Digestion Analysis

The digestion of the pCas9 plasmid using BsaI was conducted, and the results were illustrated in Figure S3. Lanes 2–5 exhibited linearized electrophoretic bands of the digested plasmid when compared with Lane 1, which served as the undigested plasmid control. The band size observed (9286 bp) corresponded with the anticipated size, thereby confirming the complete digestion of the pCas9 plasmid by BsaI.

#### 3.2.3. Validation of sgRNA Vector Construction

To generate sgRNA vectors, the forward primer pCas9-F and the reverse primer Oligo-R (which consists of antisense oligonucleotides targeting the downstream sequences) were employed for PCR amplification. The expected size of the PCR product was approximately 260 bp. The results of electrophoresis (Figure S4) indicated that all six experimental groups (Lanes 1–6) yielded amplification products measuring 269 bp, thereby confirming the successful insertion of sgRNA and the effective assembly of the recombinant plasmids.

#### 3.2.4. Dual Screening of *E. coli* DH5α Clones Containing the pCas9 Plasmid

Following transformation, colonies were selected and subjected to colony PCR to validate positive transformants. To avoid false positives arising from residual undigested or self-ligated pCas9 plasmid, a dual-primer screening strategy was employed. Initial screening used a primer pair targeting the empty pCas9 vector (pCas9-F, DR-R), which generates a 260 bp amplicon. However, in successfully constructed sgRNA-pCas9 recombinant plasmids, one primer-binding site was replaced by the sgRNA insert, preventing amplification (Figure S5). Colonies from the sgRNA-pCas9 experimental group showed no amplification (no amplification product was detected), confirming the absence of residual empty pCas9 vector. Subsequently, a second validation step was performed using primers pCas9-F and DocF-R to verify the presence of the sgRNA-pCas9 plasmid in selected colonies. PCR results confirmed that all screened colonies carried the recombinant sgRNA-pCas9 plasmid, as evidenced by amplification of the expected product (Figure S6).

### 3.3. Verification of Successful Gene Knockout

The pCas9 plasmid system was used to generate *E. coli* mutants by knocking out specific genes (*ybaV*, *rpoS*, *ycaI, ompA*, *ompR*, and *hofC*). The targeted gene regions were amplified, and the knockout outcomes were confirmed through Sanger sequencing analysis. The sequencing results for *ybaV* indicated a mutation from CCGACG to CC__CG, which involved the deletion of two base pairs in the middle, resulting in a frameshift that caused premature termination of the *ybaV* protein. Similarly, *rpoS* sequencing revealed a mutation from TCGAGA to TC__GA, also involving a two-base pair deletion. For *ycaI*, sequencing identified a point mutation (G → T) that directly converted a glutamic acid codon (GAG) to a premature stop codon (TAG). The *ompA* sequencing results demonstrated mutations from TGAAAA to TG__AA (deletion of two base pairs) and from TACAAAG to TAC__AAG. The *ompR* sequencing revealed a mutation from AGACGAG to AGATGAG, which also resulted in a premature stop codon in the downstream sequence (TAA/TAG/TGA). Lastly, *hofC* sequencing indicated a mutation from CTGATGCG to CT__CG (deletion of four base pairs) and from ATCTTT to ATTTTT (Figure S7). In comparison to the wild-type strain, the gene sequence of the knockout strain exhibited random insertion or deletion (indel) mutations near the targeted site, leading to the disruption or premature termination of the gene reading frame and consequently resulting in premature translation termination. These findings confirmed the successful mediation of gene knockout by the pCas9 system.

To confirm the successful construction of knockout strains, qPCR was performed to analyze the transcriptional levels of target genes in wild-type and six mutant strains. As shown in Figure S8, the relative expression levels of all target genes in the mutants were significantly reduced to below the detection threshold, compared with the wild type. Cycle threshold (Ct) values greater than 35, indicating no detectable amplification, suggested that transcript levels were less than 0.1% of those in the wild type. In contrast, the Ct values of the reference gene *rpoD* showed no significant differences among groups (ΔΔCt < 0.5), confirming stable RNA quality and consistent amplification efficiency. These results demonstrated that the mRNA levels of the target genes were drastically diminished or below the detection limit in the knockout strains, validating successful gene disruption.

### 3.4. Growth Curve Analysis of Mutant Strains

**Figure 3:**
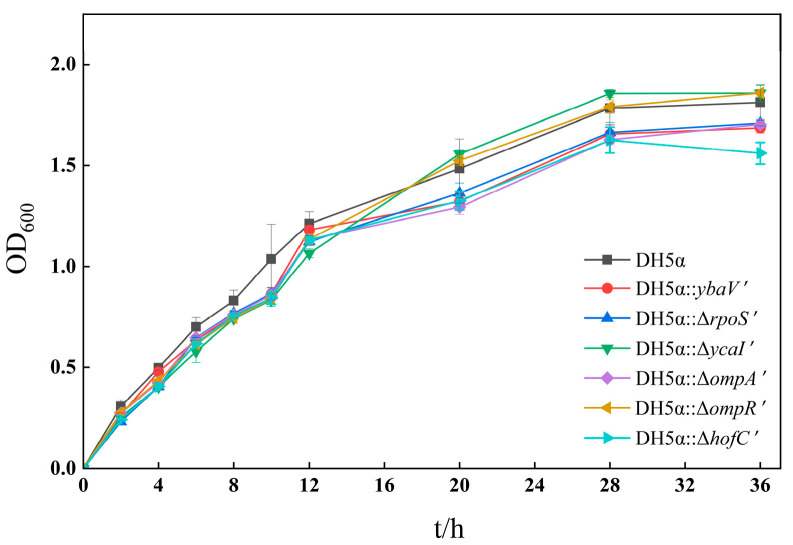
Growth curves of *E. coli* DH5α and six mutant strains.

Based on growth curve analysis (Figure 3), the wild-type and mutant strains exhibited highly consistent overall growth trends, indicating that the deletion of these genes did not significantly impair basic metabolic or proliferative capacities. During experimental measurements, the growth rates of mutant strains during the logarithmic phase were comparable to those of the wild-type strain, with no discernible differences. However, upon entering the stationary phase (16–36 h), mutants *hofC′*, *rpoS′*, *ycaI′*, and *ompA′* showed slightly reduced OD_600_ peak values compared with the wild type, indicating possible nutrient depletion or increased stress. In contrast, the growth curves of the remaining mutant strains (*ybaV′* and *ompR′*) completely overlapped with the wild type, demonstrating that their gene deletions caused no significant alterations in growth phenotypes or baseline fitness.

### 3.5. Transformation Efficiency of Mutant Strains

#### 3.5.1. Chemical Transformation Efficiency of Mutant Strains

**Figure 4:**
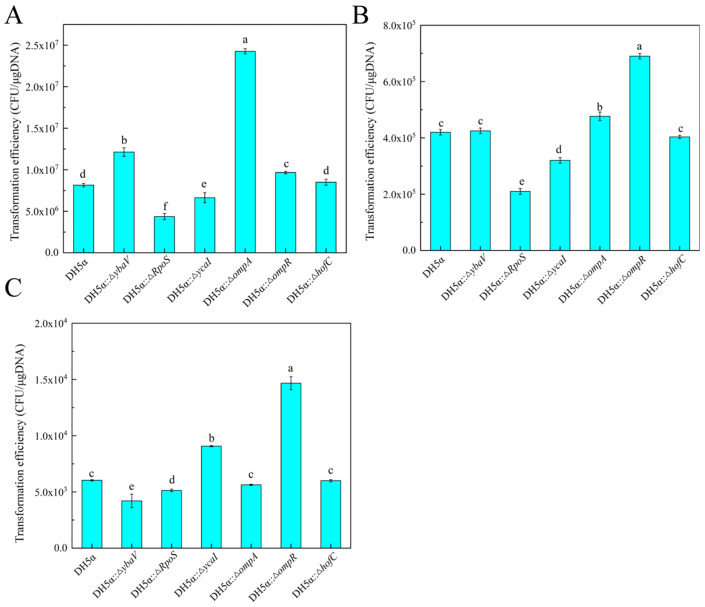
Transformation efficiency of plasmid by different mutant strain. (**A**) Chemical transformation efficiency of different mutant strains; (**B**) Ultrasonic transformation efficiency of different mutant strains; (**C**) plasmid transformation efficiency of different mutant strains. Data are presented as mean ± SD (*n* = 3 biological replicates). Statistical analysis was performed using one-way ANOVA followed by Tukey’s post hoc test (*p* < 0.05). Within each panel, bars sharing the same lowercase letter are not significantly different; different letters indicate statistically significant differences among all groups compared.

As illustrated in Figure 4A, the transformation efficiency of the mutant strains *E. coli* DH5α::*ompA′*, *ompR′* and *ybaV′* with respect to plasmid uptake was markedly superior to that of the wild-type strain. Specifically, the transformation efficiency for *ompA′* with plasmid pUC19 was recorded at 1.21 × 10^7^ CFU/μg, representing increases of 2.97 times compared with the wild-type strain (8.15 × 10^6^ CFU/μg), with statistical significance (*p* < 0.05). Conversely, the transformation efficiencies of the *rpoS′*, *ycaI′*, and mutants were found to be diminished. Notably, there was no significant difference in transformation efficiency between the *hofC′* mutant and the wild-type *E. coli* DH5α (*p* > 0.05). Furthermore, no positive transformants were observed in the negative control group treated with sterile water for both the wild-type and mutant strains. In conclusion, the deletion of the *ompA*, *ompR* and *ybaV* genes significantly enhances the chemical transformation efficiency of *E. coli* DH5α mediated by CaCl_2_, while the deletion of the *rpoS* and *ycaI* genes results in a reduction in transformation efficiency. These findings indicated that the deletion of specific genes can directly influence the transformation capabilities of bacterial competent cells.

#### 3.5.2. Ultrasound Transformation Efficiency of Mutant Strains

In previous studies, we found that the ultrasound transformation efficiency peaked at 420 W with a duration of 12 s. We investigated the ultrasound transformation efficiency of various mutant strains under this treatment, as illustrated in Figure 4B. The transformation efficiency of the *E. coli* DH5α::*ompA′* and *ompR′* mutants increased, the transformation efficiency for *ompA′* with plasmid pUC19 was recorded at 4.35 × 10^5^ CFU/μg and 6.47 × 10^5^ CFU/μg, representing increases of 1.05, 1.5 times compared with that of the wild-type strain (4.11 × 10^5^ CFU/μg), with statistical significance (*p* < 0.05). While the transformation efficiency of the *rpoS′* and *ycaI′* mutants decreased (*p* < 0.05), along with those of the *hofC′*, *ybaV′* mutants did not differ significantly from the wild-type strain (*p* > 0.05).

#### 3.5.3. Plasmid Transformation Efficiency of Mutant Strains

In the established plasmid transformation system, we investigated the plasmid transformation efficiency of various mutant strains using this method. As illustrated in Figure 4C, the plasmid transformation efficiency of the *E. coli* DH5α::*ycaI′* and *ompR′* mutants increased (*p* < 0.05), while the transformation efficiency of the *rpoS′* and *ybaV′* mutants decreased. The transformation efficiency of the *hofC′* and *ompA′* mutants did not differ significantly from that of the wild-type strains.

### 3.6. Examination of the Characteristics of Cell Membranes in Mutant Strains

#### 3.6.1. SEM Analysis of Mutant Strains

**Figure 5:**
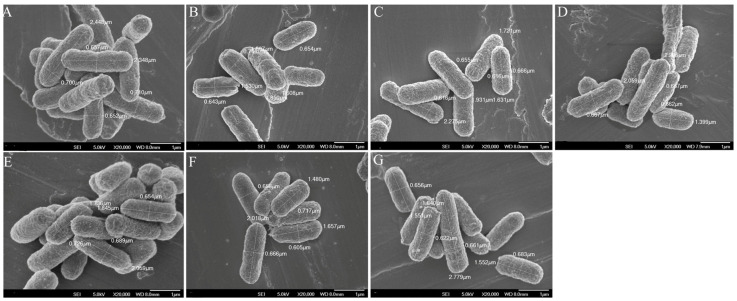
SEM observation diagram of mutant strain (setting of 3 µm, ×20000). (**A**) wild-type *E. coli* DH5α; (**B**) mutant strain *E. coli* DH5α:*ybaV′*; (**C**) mutant strain *E. coli* DH5α:*rpoS′*; (**D**) mutant strain *E. coli* DH5α: *ycaI′*; (**E**) mutant strain *E. coli* DH5α: *ompA′*; (**F**) mutant strain *E. coli* DH5α:*ompR′*; (**G**) mutant strain *E. coli* DH5α:*hofC′*.

As shown in Figure 5, the wild-type *E*. *coli* DH5α strain had a characteristic rod-shaped morphology under SEM, featuring smooth cell surfaces and complete cellular outlines. The six gene knockout mutants (*ompA′*, *ybaV′*, *rpoS′*, *ycaI′*, *ompR′*, and *hofC′*) exhibited analogous macroscopic morphology, revealing no distinguishable alterations in cell shape or surface characteristics relative to the wild-type strain (Figure 5B–G).

#### 3.6.2. AFM Analysis of Mutant Strains

**Figure 6:**
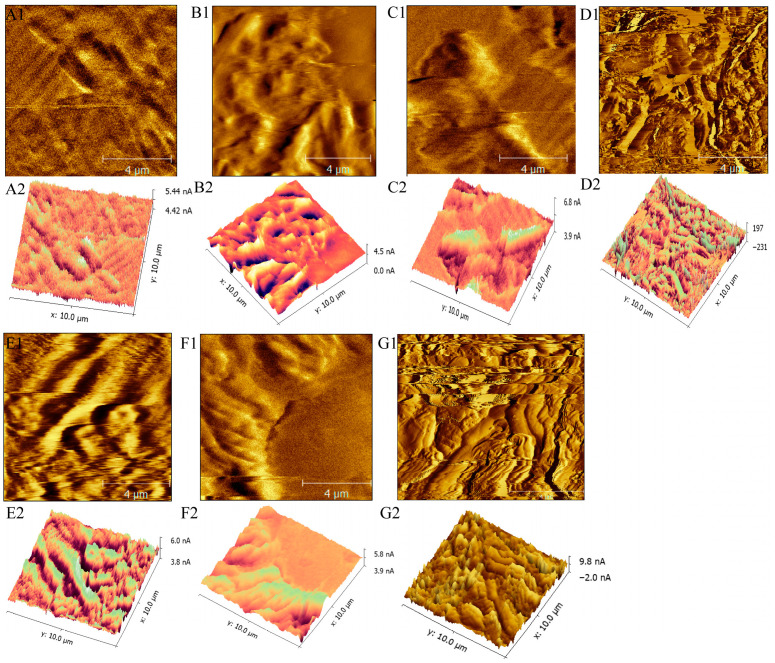
AFM phase images of *E. coli* DH5α. (**A1**–**G1**) AFM phase images of the wild-type and six mutant strains (*ybaV′*, *rpoS′*, *yca*I*′*, *ompA′*, *ompR′*, *hofC′*). (**A2**–**G2**) Corresponding AFM three-dimensional height maps of the identical fields of view shown in (**A1**–**G1**). Scan area: 10 μm × 10 μm.

Atomic force microscopy (AFM) was additionally utilized to elucidate the surface topography of *E*. *coli* DH5α with enhanced spatial resolution. Figure 6 illustrates that untreated wild-type cells displayed a characteristic rod-shaped morphology, characterized by a relatively smooth surface and uniform height distribution. In comparison to the wild-type strain, the six gene knockout mutants (*ybaV′*, *rpoS′*, *ycaI′*, *ompA′*, *ompR′*, and *hofC′*) exhibited analogous overall surface properties and cellular dimensions (Figure 6(B1–G1,B2–G2)). Nonetheless, the identified commonalities were limited to broad physical characteristics and do not exclude more nuanced nanoscale variations.

#### 3.6.3. Conductivity of Mutant Strains

**Figure 7:**
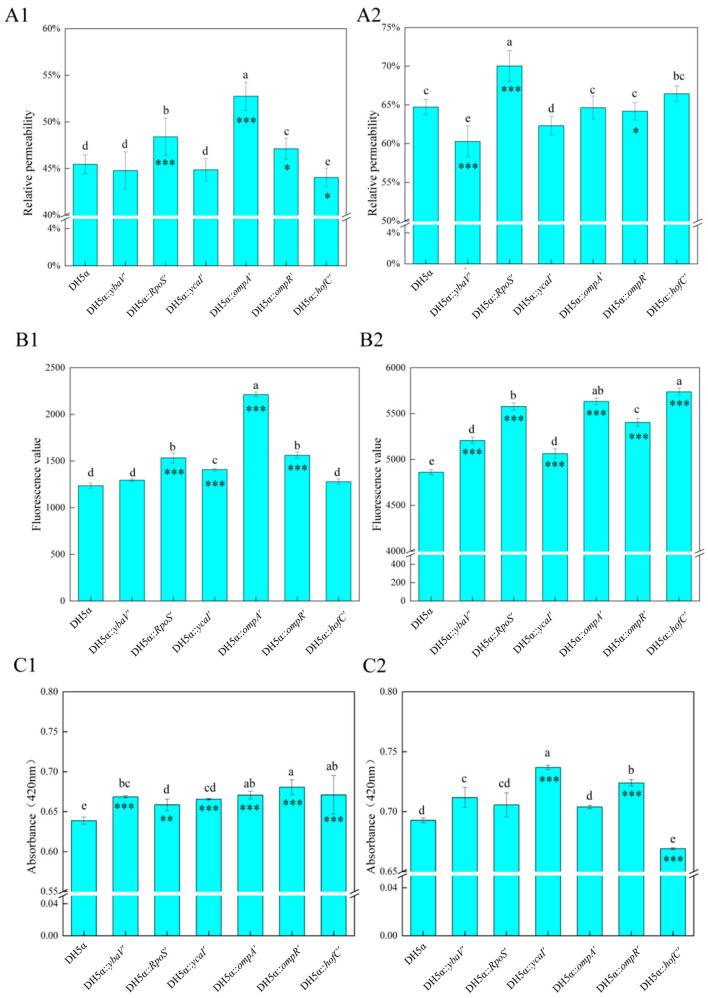
Chart for the assessment of membrane permeability index. (**A1**) Graphical representations of electrical conductivity measurements for both normal wild-type and mutant strains of *E. coli*. (**A2**) A plot illustrating the conductivity measurements over a duration of 12 s for wild-type and mutant *E. coli* subjected to 300 W ultrasonic treatment. (**B1**) PI fluorescence measurement diagrams of normal wild-type and mutant *E. coli*. (**B2**) PI fluorescence assay plots after 300 W ultrasonic treatment of wild-type and mutant *E. coli* for 12 s. (**C1**) ONPG fluorescence assay diagrams of normal wild-type and mutant *E. coli*. (**C2**) ONPG fluorescence assay plots after 300 W ultrasonic treatment of wild-type and mutant *E. coli* for 12 s. Data are mean ± SD (*n* = 3). Different lowercase letters above bars indicate statistically significant differences among all groups as determined by one-way ANOVA with Tukey’s post hoc test (*p* < 0.05). Asterisks above specific bars indicate significant differences compared to the wild-type control within the same condition, assessed by Student’s *t*-test: * *p* < 0.05, ** *p* < 0.01, *** *p* < 0.001.

As illustrated in Figure 7(A1), the relative conductivity of the wild-type *E. coli* DH5α strain was 45.45%. The *rpoS′*, *ompA′*, and *ompR′* mutants demonstrated a statistically significant increase in conductivity (*p* < 0.05), indicating enhanced ion leakage consistent with increased membrane damage. In contrast, the *ybaV′* and *ycaI′* mutants showed no significant differences (*p* > 0.05), while the *hofC′* mutant displayed a slight decrease in conductivity. Notably, *ompA′*, which exhibited the highest transformation efficiency (as discussed in Section 3.6), also presented the most substantial increase in conductivity, supporting the hypothesis that increased membrane permeability facilitates transformation. However, the conductivity patterns observed in *ybaV′* (which had elevated transformation efficiency) and *rpoS′, ompR′* (which had reduced transformation efficiency) were unexpected and merit further investigation. In the context of ultrasonication (as depicted in Figure 7(A2)), all strains exhibited increased conductivity relative to untreated controls. The *rpoS′* mutant displayed the most significant increase, likely attributable to compromised stress-response mechanisms, while *ycaI′* and *ybaV′* showed smaller increases. The other mutants (*ompA′*, *ompR′*, *hofC′*) did not demonstrate significant deviations from the wild-type strain, which may suggest complete membrane disruption as a result of ultrasonication.

#### 3.6.4. PI Staining for Membrane Permeability in Mutants

As illustrated in Figure 7(B1), propidium iodide (PI) staining demonstrated variations in fluorescence intensity that correlate with membrane integrity. The wild-type strain *E. coli* DH5α exhibited a baseline fluorescence level of 1273. Among the mutant strains, *ompA′* displayed the highest fluorescence intensity at 2229, representing a 1.75-fold increase, The mutants *ycaI′*, *rpoS′*, *ompR′* showed a modest increase in fluorescence, while *ybaV′* and *hofC′* exhibited no significant alterations, suggesting that the deletions of these genes have a minimal impact on membrane integrity.

In the context of ultrasonication (Figure 7(B2)), all strains demonstrated increased fluorescence due to membrane disruption. The mutants *ompA′*, *rpoS′*, *ompR′*, and *hofC′* exhibited substantial increases in fluorescence. This observation highlights the combined effects of gene deletion and ultrasonic stress on membrane permeability. Notably, while *hofC′* did not show significant changes in fluorescence without ultrasonication, it exhibited considerable permeability following treatment, suggesting that membrane vulnerability is context-dependent. These findings confirmed that deletions of *ompA*, *rpoS*, and *ompR* genes significantly enhance membrane permeability, particularly under conditions of stress.

#### 3.6.5. ONPG Permeability Assay

As shown in Figure 7(C1), wild-type *E. coli* DH5α exhibited an absorbance of 0.641. Among mutants, *ompA′* and *ompR′* showed the highest absorbance values, suggesting increased IM permeability facilitating ONPG entry or enzymatic hydrolysis. The mutants *hofC′* and *ybaV′* displayed minor absorbance increases, while the mutants *ompA′* and *ycaI′* had values comparable to the wild type. Notably, the elevated absorbance of *ompR′* may reflect defective IM protection mechanisms. Under ultrasonication (Figure 7(C2)), all mutants except *hofC′* showed slight absorbance increases. *ycaI′* exhibited a marked post-ultrasonication fluorescence surge (mechanism unclear), while the mutants *ompA′*, *rpoS′*, and *ompR′* demonstrated significant IM permeability enhancement, consistent with their conductivity and transformation efficiency profiles. These results further validate *ompA*, *rpoS*, and *ompR* genes as critical regulators of membrane dynamics under stress.

### 3.7. Establishment of Kinetic Model Simulation for the Mutant Strain E. coli DH5α::ompA′

#### 3.7.1. The Establishment of the Membrane Permeability Model

According to the law of conservation of mass and the assumptions made, the following can be derived:

(1) $$\frac{dE}{dt}=-k_{1}-k_{2}E+k_{3}F$$

The parameter k is independent of the variables t, E, and F, but it is related to ultrasonic intensity and frequency. The relationship between F, E, and T is expressed as F = T − E, where T represents the total amount of intracellular and extracellular permeating substances. Substituting this relationship into Equation (1) yields the following result:

$$E(t)=y,\;b=-(k_{2}+k_{3}),\;a=\frac{k_{2}T-k_{1}}{k_{2}+k_{3}},\;c=\frac{k_{3}T-k_{1}}{k_{2}+k_{3}}$$

(2) $$E(t)=\frac{k_{2}T-k_{1}}{k_{2}+k_{3}}\cdot e^{-(k_{2}+k_{3})t}+\frac{k_{3}T-k_{1}}{k_{2}+k_{3}}$$

The relationship between the concentration of intracellular substances and time can be simulated as follows:

(3) $$y={ae}^{bt}+c$$

By utilizing the fluorescence probe method, we observed changes in the fluorescence intensity of FDA over time in *E. coli* treated with specific ultrasonic power. Data analysis was conducted, and a kinetic model was simulated to interpret the experimental measurement results. This process derived a fitting equation for the concentration of intracellular permeable substances over time after ultrasonic treatment at different power levels.

#### 3.7.2. Alterations in Cell Membrane Permeability and Kinetic Model Simulation

**Figure 8:**
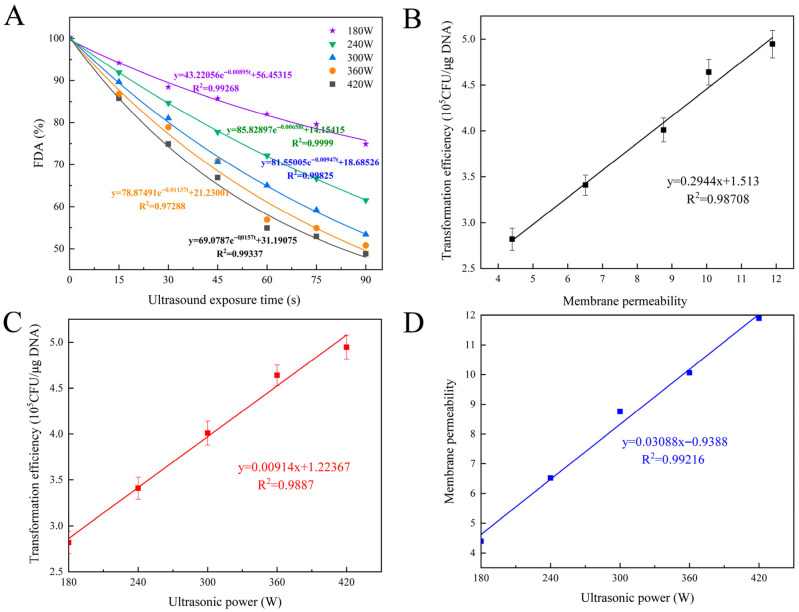
Experimental values and permeability model simulations of FDA under ultrasonication and relationship between transformation efficiency, membrane permeability and ultrasonic power of mutant strain *E. coli* DH5α:*ompA′*. Note: (**A**) Experimental values and permeability model simulations of FDA under ultrasonication. (**B**) Fitting equation of transformation efficiency and membrane permeability. (**C**) Fitting equation of ultrasonic power and transformation efficiency. (**D**) Fitting equations of ultrasonic power and membrane permeability.

To evaluate the compatibility of the knockout strains with the previously established model, a simulation of the permeability model for the mutant strain *E. coli* DH5α:*ompA′* was conducted. The experimental data presented in Figure 8A indicated that after staining the ultrasound-treated cells with FDA, fluorescence spectrophotometry revealed a significant decline in the FDA fluorescence labeling rate of the cells as the duration of ultrasound exposure increased (*p* < 0.05). Further analysis demonstrated a positive correlation between the ultrasonic power parameters and the rate of fluorescence attenuation, which aligns with the observed trends in the normal strains. For instance, the percentage of FDA fluorescence was recorded at 74.89% when 180 W of ultrasound was applied for 90 s. Conversely, when the ultrasonic power was increased to 420 W for the same duration, the FDA fluorescence percentage decreased to 48.8% [20].

The numerical simulation analysis of the experimental data, based on the kinetic model outlined in Equation (3), demonstrated that under varying ultrasonic power conditions, the FDA fluorescence intensity curve exhibits a strong correlation with the exponential model (R^2^ ≥ 0.98). This finding supported the effectiveness of the kinetic equation in characterizing the changes in cell membrane permeability induced by ultrasound in *E. coli*. Additionally, it was confirmed that the membrane permeability of the mutant strain aligns with the previously established equation. The theoretical framework developed in this study provided a quantifiable mathematical model that serves as a foundation for analyzing the dynamic response mechanisms of microbial cell membranes to ultrasound exposure.

#### 3.7.3. Modeling the Quantitative Relationship Among Cell Membrane Permeability, Ultrasonic Power, and Transformation Efficiency

The penetration of substances into *E. coli* cell membranes occurred simultaneously with ultrasonic action. By taking the derivative of Equation (3), Equation (4) can serve as an instantaneous membrane permeability evaluation index. Integrating Equation (4) provided a measure of membrane permeability over a specific time interval.

(4) $$\frac{dE}{dt}=abe^{bt}$$

(5) $$\int_{0}^{t}\frac{dE}{dt}dt=\int_{0}^{t}abe^{bt}dt={\left.ae^{bt}\right|}_{0}^{t}$$

By quantifying the membrane permeability under different ultrasonic power levels using Equation (5) and combining it with transformation experiments of *E. coli* under ultrasonic treatment, a quantitative relationship model between transformation efficiency, membrane permeability, and ultrasonic power within a specific range of ultrasonic power was inferred and fitted.

#### 3.7.4. Quantitative Relationship Model of Ultrasound Power, Membrane Permeability, and Transformation Efficiency in the Mutant Strain *E. coli* DH5α:ompA′

The established kinetic model elucidated the relationship between the intracellular concentrations of permeable substances, ultrasonic power, and duration while also detailing the dynamics of alterations in membrane permeability. Equation (4) serves as an evaluative index for transient membrane permeability and is integrated over a duration of 12 s of ultrasonic exposure. Utilizing the parameters (a), (b), and Formula (5) depicted in Figure 8A, the membrane permeability of the ultrasonic-treated mutant strain *E. coli* DH5α:*ompA′* was calculated over the 12 s interval (refer to Table 1).

An analysis of membrane permeability under varying ultrasonic power treatments reveals a correlation between membrane permeability and ultrasonic power, leading to the formulation of a fitting equation. As illustrated in Figure 8, the fitting equation representing the relationship between transformation efficiency and membrane permeability was expressed as: (y = 0.0294x + 1.513) (R^2^ = 0.9871) (Figure 8B). Additionally, the fitting equation for transformation efficiency as a function of ultrasonic power was expressed as: (y = 0.0092x + 1.2237) (R^2^ = 0.9887) (Figure 8C). Furthermore, the fitting equation relating membrane permeability to ultrasonic power in the context of transformation efficiency was expressed as: (y = 0.0309x + 0.9388) (R^2^ = 0.9922) (Figure 8D). Notably, the correlation coefficients for these equations exceed 0.97 within a specified range. Within a defined ultrasonic power range, the quantitative relationship model between transformation efficiency and membrane permeability for the mutant *E. coli* DH5α:*ompA′* adhered to the form (T = aP + c) (where (a) and (c) are constants, (T) represents transformation efficiency, and (P) denotes membrane permeability), thereby confirming the consistency of the constructed model [20].

### 3.8. FTIR Analysis

**Figure 9:**
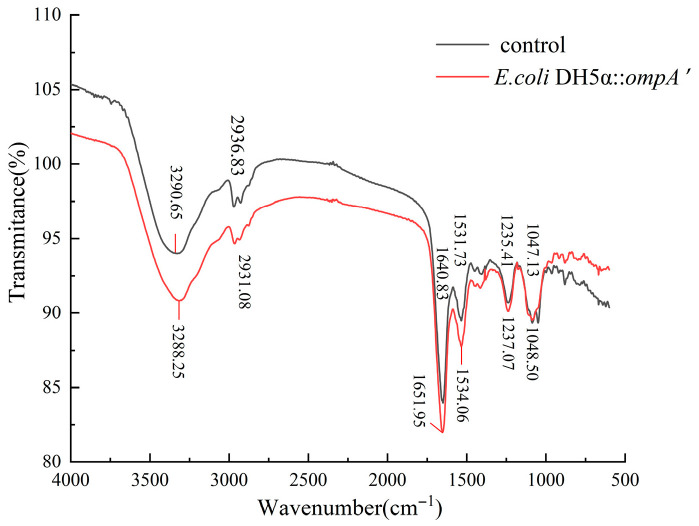
FTIR spectral analysis of *E. coli* DH5α wild-type and DH5α::*ompA′* mutant strain.

As shown in Figure 9, In the 3500–3000 cm^−1^ range, the wild-type strain had a prominent absorption peak at 3290.65 cm^−1^, whereas the mutant strain presented a similar feature peak at 3288.55 cm^−1^. This region is generally linked to the stretching vibrations of O–H or N–H bonds. The noted discrepancies in peak position and intensity indicate that the two strains may exhibit minor variances in the makeup of hydrogen-containing functional groups in biomolecules or in their hydrogen-bonding milieu [40]. At around 2900 cm^−1^, both strains exhibited characteristic C–H stretching vibration absorption peaks (wild-type at 2936.83 cm^−1^, mutant at 2931.08 cm^−1^). The relative locations and intensity variations in these peaks may indicate nuanced alterations in the overall cellular composition of lipids or carbohydrates [18]. In the 1600–1500 cm^−1^ range, associated with the amide I and amide II bands, notable discrepancies in peak locations and intensities were detected between the wild-type and mutant strains (1640.83/1531.73 cm^−1^ versus 1651.95/1534.06 cm^−1^). This region predominantly results from protein backbone vibrations, suggesting that these variances may correlate with changes in overall protein structure or microenvironment [41,42]. Moreover, the absorption peaks within the 1200–1000 cm^−1^ range primarily correspond to C–O linked vibrations in polysaccharides or nucleic acids. Their changes indicate that the mutant strain may have experienced modifications in its overall cellular chemical composition [43].

## 4. Discussion

Bacterial genetic transformation is a multifaceted process governed by numerous factors, encompassing aspects such as membrane structural characteristics, regulatory networks, and external environmental influences [44,45]. While membrane permeability has traditionally been viewed as a crucial factor in DNA absorption efficiency, growing evidence suggests that variations in membrane barrier characteristics alone cannot entirely explain the discrepancies in transformation efficiency [5,45]. In our prior research, we developed a mathematical model delineating the correlation between membrane permeability and transformation efficiency (T = aP + c), which offered a vital framework for comprehending transformation [20]. This approach does not elucidate which host genes regulate the crucial variable (P) or whether they also affect transformation via other independent pathways. This work conducted functional validation to investigate the functions of candidate genes in influencing membrane permeability and transformation efficiency via direct genetic disruption. The findings indicated that deletions of various genes produced significantly diverse impacts on membrane permeability and transformation efficiency, with no straightforward one-to-one correlation between the two. This conclusion suggests that while the previously developed model has predictive validity, it is unable to completely clarify the fundamental mechanisms governing transformation regulation.

The mutant strains created with CRISPR-Cas9 gene editing exhibited a deletion of *ompA*, which markedly enhanced membrane permeability and correlated with an increase in transformation efficiency, aligning with Ambrosi’s findings [46]. This underscores the essential function of *ompA* in preserving the integrity of the OM barrier. Nonetheless, the impact of *ompR* deletion on membrane permeability was not wholly congruent with that of *ompA*, despite both being linked to OM control. *OmpR*, as a worldwide transcriptional regulatory factor, regulates the expression of many porins and stress-related genes and may affect the transformation process via intricate regulatory pathways [47]. The deletion of *ycaI* significantly diminished transformation efficiency without noticeably affecting membrane permeability, indicating that this gene likely plays a role in structures or processes associated with DNA uptake rather than directly influencing membrane barrier function during transformation [48]. Likewise, *ybaV* exhibits sequence homology with the natural competence protein ComEA [49]. The loss adversely impacts transformation efficiency but does not significantly alter membrane permeability, thereby reinforcing its possible function in DNA recognition, binding, or transport. The role of the global stress regulating factor *rpoS* is also indicative [50]. The absence of *rpoS* results in heightened membrane permeability, while transformation efficiency significantly declines. The reverse effect indicates that stress response pathways may significantly impact the transformation process by modulating DNA-binding ability, intracellular DNA processing, or the coordinated mechanisms between the OM and IM [51]. These findings collectively suggest that the regulation of transformation in *E*. *coli* is not controlled by a singular factor but rather involves a complex regulatory system comprising membrane structure proteins, DNA uptake-related components, and overarching regulatory networks [20,52]. Utilizing data from the *ompA′* mutant strains, we further validated the accuracy of the previously developed model. Moreover, under conditions of chemical transformation, ultrasound-mediated transformation, and plasmid co-incubation transformation, many mutant strains demonstrated unique response patterns. This indicates that the molecular process of DNA uptake may alter across various transformation contexts and that functional relationships or competition among membrane proteins may also influence the efficiency of DNA transfer [53]. These hypotheses will undergo further exploration and validation in subsequent investigations.

The conclusions of this work are derived not from the absolute phenotype of a singular mutant strain but from consistent trends identified across many gene knockout strains, diverse transformation techniques, and various indicators of membrane permeability. Consequently, although certain CRISPR-Cas9-generated mutants may display undesired off-target effects, their impact on the overarching conclusions is minimal. Off-target effects generally induce random and strain-specific perturbations, making it improbable to yield consistent gene-associated response patterns across varying transformation settings [54]. The alterations noted in transformation efficiency and membrane characteristics among the mutant strains in this study exhibited a significant level of consistency across independent experiments, suggesting that these trends predominantly represent the functional outcomes of specific gene deletions. This study elucidates the potential functions of genes, including *rpoS*, *ompA*, *ompR*, *ycaI*, *hofC*, and *ybaV*, in preserving OM integrity, improving environmental adaptability, and modulating plasmid transformation efficiency, thereby offering novel insights into the mechanisms that are not yet fully understood in gene function research. Moreover, our findings provide an empirical basis and theoretical direction for enhancing gene editing technology, increasing transformation efficiency, and creating highly efficient designed strains.

This study validated the applicability of a previously established linear model connecting membrane permeability to transformation efficiency by knocking out six membrane-related genes and conducting phenotypic comparisons under three transformation conditions. Additionally, it uncovered a more profound regulatory logic, particularly concerning key gene mutations such as *ompA*. Transformation efficiency is not solely governed by a single gene that modifies membrane permeability; instead, it is collectively regulated by a complex, condition-dependent genetic network. Experiments demonstrated that the elimination of specific genes, such as *ycaI*, *ybaV*, and *rpoS*, might markedly affect transformation efficiency without substantially modifying membrane permeability, suggesting the presence of parallel regulatory pathways that operate independently of this linear model. This study accurately defined the strict limits of validity for the quantification model through targeted genetic perturbations. The concept is exclusively relevant under particular sonication settings inside the *ompA* deletion context. This discovery concurrently signifies the presence of more intricate underlying genetic regulation.

## 5. Conclusions

This study utilized CRISPR-Cas9 gene knockout technology to investigate the functions of genes including *rpoS*, *ompA*, *ompR*, *ycaI*, *hofC*, and *ybaV* in *E. coli* membrane homeostasis, stress response, and plasmid transformation. The creation and examination of *ompA* deletion mutant strains produced results that corroborated the validity of the previously suggested model connecting membrane permeability and transformation efficiency at the genetic level. In scenarios involving chemical transformation, ultrasound-mediated transformation, and plasmid incubation-based transformation conditions, diverse mutant strains exhibited unique transformation response patterns. The findings indicate that double-stranded DNA may utilize distinct uptake mechanisms during natural and artificial transformation, while functional connections or competitive dynamics among membrane-associated proteins could influence transformation efficiency. Nevertheless, the fundamental mechanisms necessitate additional inquiry for validation. This study presents experimental observations regarding the roles of various membrane-associated and regulatory genes in the plasmid transformation process of *E. coli*, thus providing a foundational reference for future functional analyses of these genes and the optimization of transformation conditions.

## Figures and Tables

**Table 1:** Membrane permeability corresponding to different ultrasonic powers during ultrasonic treatment for 12 s.

Ultrasonic Power	Equation	R^2^	Membrane Permeability
180 W	y = 43.2206e^−0.0090t^ + 56.4532	R^2^ = 0.9927	4.4013
240 W	y = 85.8290e^−0.0066t^ + 14.1542	R^2^ = 0.1000	6.5156
300 W	y = 81.5501e^−0.0095t^ + 18.6853	R^2^ = 0.9983	8.7601
360 W	y = 78.8749e^−0.0114t^ + 21.2300	R^2^ = 0.9729	10.0598
420 W	y = 69.0787e^−0.0157t^ + 31.1908	R^2^ = 0.9865	11.8942

## Data Availability

The original contributions presented in this study are included in the article/Supplementary Materials. Further inquiries can be directed to the corresponding author.

## Supplementary Materials

The following supporting information can be downloaded at: https://www.mdpi.com/article/10.3390/microorganisms14010198/s1, Figure S1: Cell membrane permeability model; Figure S2: (A) Gene interaction network diagram; (B) Chromosome localization analysis diagram; Figure S3: Restriction enzyme digestion analysis of the pCas9 plasmid; Figure S4: Agarose gel electrophoresis of PCR analysis for recombinant plasmids; Figure S5: Agarose gel electrophoresis of PCR analysis for sgRNA-pCas9 recombinant plasmids transformed into *E. coli*; Figure S6: Detection of sgRNA-pCas9 positive recombinants by specific strains; Figure S7: Sequence alignment maps of gene mutation sites; Figure S8: qPCR expression levels in gene knockout and control groups of cells; Table S1: Table of sgRNA primer design; Table S2: Phosphorylation reaction components and conditions for oligonucleotide primers; Table S3: Restriction digestion system for the pCas9 plasmid and ligation system for constructing recombinant vectors; Table S4: Primer design and reaction setup for colony PCR; Table S5: Genes relevant to cell membrane in *Escherichia coli*; Table S6: Differences among transformation systems; Table S7: Selected knockout genes and functional descriptions.
