# Supplementary material for: Functional Analysis of CRISPR-Cas9-Mediated Gene Deletion in E. coli DH5α on Membrane Permeability and Transformation Efficiency
Source: Microorganisms. 2026 Jan 15;14(1):198. doi: 10.3390/microorganisms14010198 (PMC12844161; doi:10.3390/microorganisms14010198)
Supplement: Supplementary file 1 [file microorganisms-14-00198-s001.zip › microorganisms-4041174-supplementary.pdf]

Supplementary Information

Table S1. Table of sgRNA primer design

Gene	Primer Sequence (5'-3')
sgRNA1-rpoS-F	AAACAGTTACTCTCGATCATCCGGG
sgRNA1-rpoS-R	AAAACCCGGATGATCGAGAGTAACT
sgRNA2-rpoS-F	AAACGGTCAAACTTCTCTACCGCGG
sgRNA2-rpoS-R	AAAACCGCGGTAGAGAAGTTTGACC
sgRNA1-ompA-F	AAACTGTAAGCGTCAGAACCGATGG
sgRNA1-ompA-R	AAAACCATCGGTTCTGACGCTTACA
sgRNA2-ompA-F	AAACGGTTCTGACGCTTACAACCAG
sgRNA2-ompA-R	AAAACTGGTTGTAAGCGTCAGAACC
sgRNA1-ompR-F	AAACCAAGGTTCATCAGCTTATCGG
sgRNA1-ompR-R	AAAACCGATAAGCTGATGAACCTTG
sgRNA2-ompR-F	AAACGGAACATTTCGCGCGTACCGG
sgRNA2-ompR-R	AAAACCGGTACGCGCGAAATGTTCC
sgRNA1-hofC -F	AAACATTATGGGTTCACTGATGCGG
sgRNA1-hofC -R	AAAACCGCATCAGTGAACCCATAAT
sgRNA2-hofC -F	AAACTACCGGTCAGTTCACCCGTGG
sgRNA2-hofC -R	AAAACCACGGGTGAACTGACCGGTA
sgRNA1-ycaI-F	AAACTACCACCAGTAAGAACCATGG
sgRNA1-ycaI-R	AAAACCATGGTTCTTACTGGTGGTA
sgRNA2-ycaI-F	AAACCATGAACATCTGGATCACCGG
sgRNA2-ycaI-R	AAAACCGGTGATCCAGATGTTCATG
sgRNA1-ybaV-F	AAACTTCTGTGGCGAAACCGACGGG
sgRNA1-ybaV-R	AAAACCCGTCGGTTTCGCCACAGAA
sgRNA2-ybaV-F	AAACGCTGGTGGAACGTAATCTGGG
sgRNA2-ybaV-R	AAAACCCAGATTACGTTCCACCAGC

Table S2. Phosphorylation reaction components and conditions for oligonucleotide primers

Component	Volume (μL)	Reaction Conditions
Forward oligo (10 μmol/L)	5	1. 95ºC, 5:00 (denaturation);<br>2. 95ºC, 1:30, -1ºC per cycle;<br>3. Go to Step 2, 70X;<br>4. 4ºC, ∞ (annealing).
Reverse oligo (10 μmol/L)	5	
ddH_2O	8	
Annealing buffer (10×)	2	
Total volume	50	

Table S3. Restriction digestion system for the pCas9 plasmid and ligation system for constructing recombinant vectors

Component	Volume
Substrate DNA	≤1 μg
10 × Buffer BsaI	2 μL
BsaI	0.5-1 μL
Ultrapure water	To 20 μL

Component	Volume
Digested pCas9	50-100 ng
Insert DNA	Approximately 3-fold molar excess of insert relative to vector
10 × Ligation buffer	2 μL
T4 DNA ligase	1 μL
ddH_2O	20 μL

Table S4. Primer design and reaction setup for colony PCR

Component	Volume (µL)	Amplicon Size (bp)	Reaction Conditions
Primer F (10 µmol/L)	1	269	Initial Denaturation: 94°C for 30 s.
Primer R (10 µmol/L)	1	269	Cycling Steps (30 cycles):
2 × pro Taq Master	25	269	- Denaturation: 98°C for 10 s.
Template DNA	1 (<500 ng)	269	- Annealing: 55°C for 30 s.
ddH_2O	To 50	269	- Extension: 72°C for 1 min.<br>Final Extension: 72°C for 2 min.

Primer	Primer Sequence (5’-3’)
pCas9-F-primer	AACACGCATTGATTTGAGTCAGCTA
DocF	GAAACAAGCGCTCATGAGCCCG
DR-R	GAGACCTTTGAGCTTCCGAGACTGGTCTCA

Table S5. Genes relevant to cell membrane in Escherichia coli

Gene	Primer Sequence (5’-3’)
Q-ybaV-F	CGGCGATAAACAGCTGGATT
Q-ybaV-R	TTCGCCAGCATCAGTTTACC
Q-rpoS-F	CGCGTTGATGAAATCGCTAC
Q-rpoS-R	TTGCCAGCCAGTTTACCGTA
Q-ycaI-F	ATGCGCCGTAAACAGGATCT
Q-ycaI-R	TCCGGCGATTACCTGTTCTT
Q-ompA-F	GCTGGTGGTGCGATATTGCT
Q-ompA-R	CGCCAGACGATACCGAACAT
Q-ompR-F	GATCCGCTGCTGAAAGACGA
Q-ompR-R	CAGGCGGTAAACGCATTGAT
Q-hofC -F	TGAACGCCTGAACGAACTTC
Q-hofC -R	ATCGGCGACGAACTTGTTCT
Q-rpoD-F	CGCGATGAAGTTCAACGACCT
Q-rpoD-R	TTCGGCGTTGATGTTCTTGG

Table S6 Differences among transformation systems

Transformation system	Buffer/Solution	Core processing steps
Chemical transformation	100 mmol/L $Cacl_2$ (ice-cold)	Ice bath incubation: 30 min<br>Heat shock: 90 s (42°C)<br>Recovery: 1 (h37°C, shaking)
Ultrasound-mediated transformation	0.1mol/L $Cacl_2$ (ice-cold)	Ultrasonic treatment: 12 s (40 kHz, 300 W)<br>Recovery: 1 h (37°C, static)
Plasmid incubation-based transformation condition	LB medium + 100mmol/L $Cacl_2$	Only Ca^{2+} present, no vigorous treatment

Table S7. Selected knockout genes and functional descriptions

Gene	Gene description
ybaV	Putative competence-suppressing periplasmic helix-hairpin-helix DNA-binding protein.
rpoS	RNA polymerase sigma factor rpoS; This sigma factor is the master transcriptional regulator of the stationary phase and the general stress response, which are mainly involved in metabolism, transport, regulation and stress management.
ycaI	ComEC family inner membrane protein.
ompA	Outer membrane protein A; With TolR probably plays a role in maintaining the position of the peptidoglycan cell wall in the periplasm (Probable). Plays a role in resistance to environmental stress.
ompR	DNA-binding transcriptional dual regulator *ompR*.
hofC	Assembly protein in type IV pilin biogenesis, transmembrane protein; Putative integral membrane protein involved in biogenesis of fimbriae, protein transport, DNA uptake.

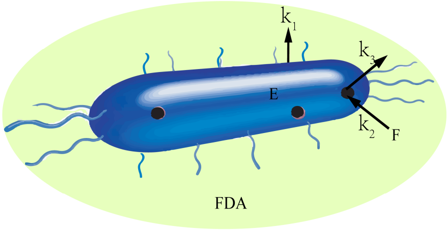

Figure S1. Cell membrane permeability model. E represented concentration of intracellular fluorescein; F represented concentration of extracellular fluorescein; k_1 represented rate constant of fluorescein loss caused by cell death (constant value); k_2 represented rate constant of fluorescein permeating from extracellular to intracellular space (constant value); k_3 represented rate constant of fluorescein permeating out of intracellular and then re-permeating into intracellular space (constant value). For the materials and ultrasound equipment used, k_1, k_2, k_3, and T are all constant values.

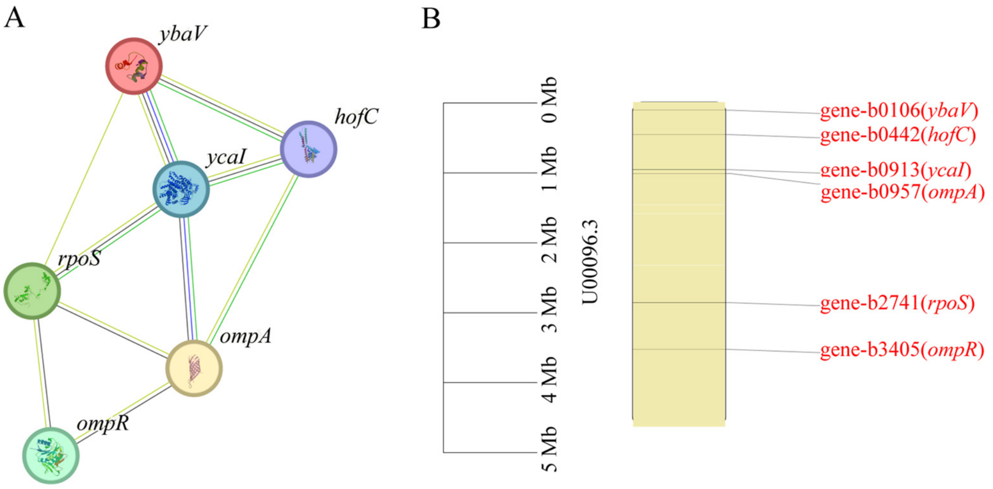

Figure S2. (**A**) Gene interaction network diagram; (**B**) Chromosome localization analysis diagram

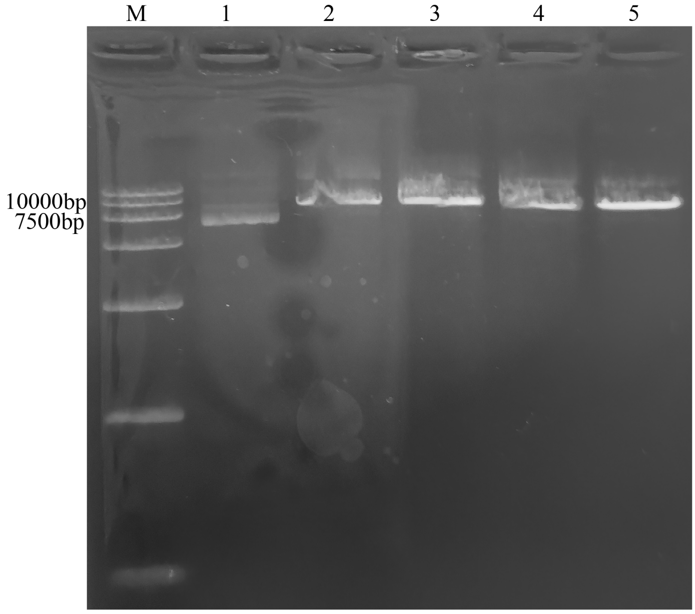

Figure S3. Restriction enzyme digestion analysis of the pCas9 plasmid

Note: Lane M: DNA Ladder Marker; Lane 1: pCas9 plasmid; Lanes 2-5: Products of pCas9 plasmid digested with BsaI.

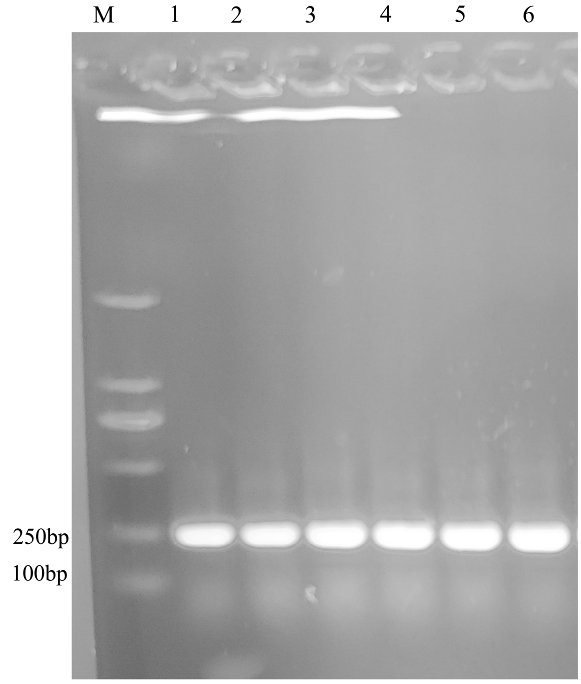

Figure S4. Agarose gel electrophoresis of PCR analysis for recombinant plasmids

Note: Lane M: DNA Ladder Marker; Lanes 1-3: PCR products of the sgRNA-pCas9 recombinant plasmids constructed with ybaV, rpoS, and ycaI genes, respectively; Lanes 4-6: PCR products of the sgRNA-pCas9 recombinant plasmids constructed with ompA, ompR, and hofC genes, respectively.

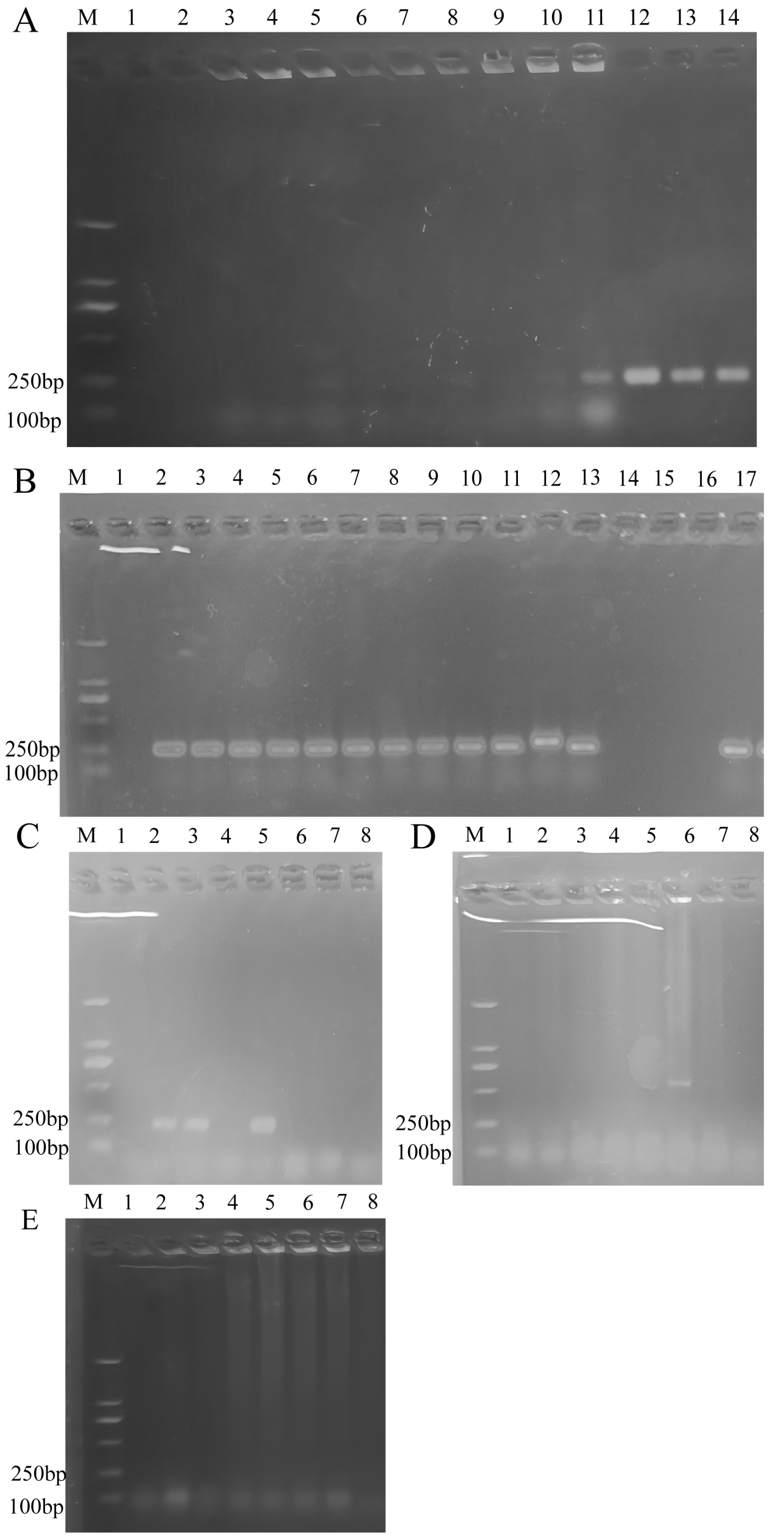

Figure S5. Agarose gel electrophoresis of PCR analysis for sgRNA-pCas9 recombinant plasmids transformed into E. coli

Note: Lane M: 2000 bp DNA Ladder Marker. Figure A (Lanes 1-7 and Lanes 8-14): PCR analysis of sgRNA-pCas9 recombinant plasmids transformed into E. coli for ybaV and rpoS gene constructs, respectively. Figures B, C, D, E: PCR analysis of sgRNA-pCas9 recombinant plasmids transformed into E. coli for ycaI, ompA, ompR, and hofC gene constructs, respectively.

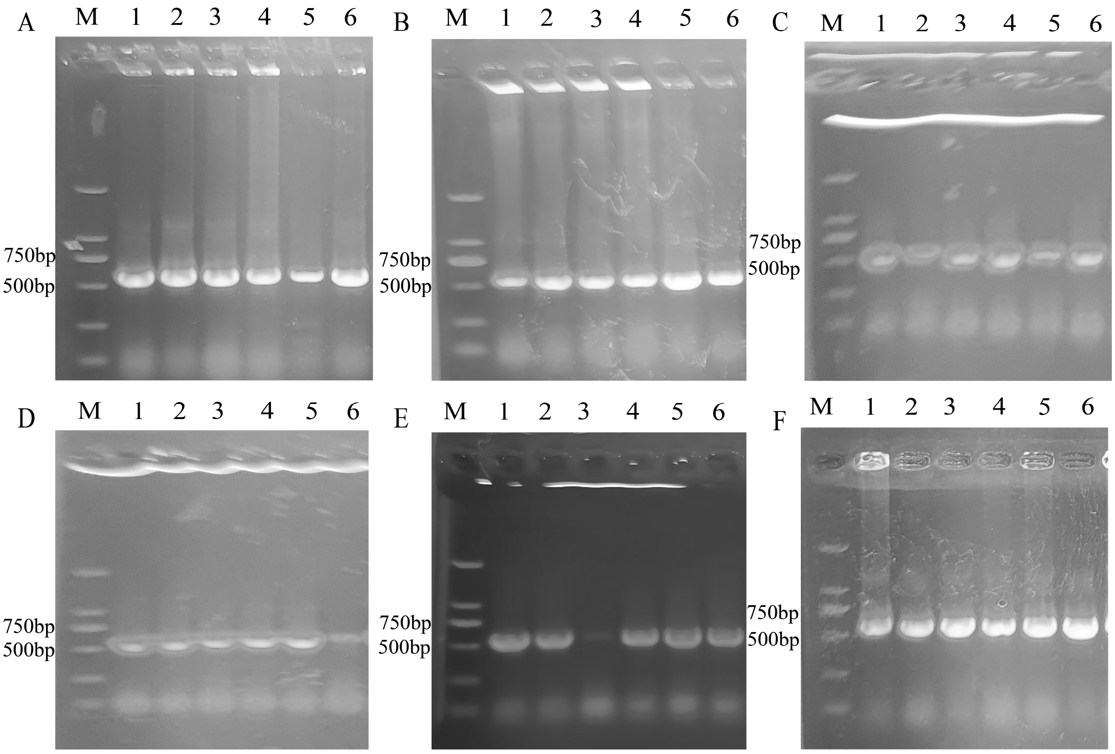

Figure S6. Detection of sgRNA-pCas9 positive recombinants by specific strains

Note: Lane M: 2000 bp DNA Ladder Marker; Lanes A1-A6: E. coli DH5α:ybaV’ positive recombinants; Lanes B1-B6: E. coli DH5α:rpoS’positive recombinants; Lanes C1-C6: E. coli DH5α:ycaI’ positive recombinants; Lanes D1-D6: E. coli DH5α:ompA’ positive recombinants; Lanes E1-E6: E. coli DH5α:ompR’ positive recombinants; Lanes F1-F6: E. coli DH5α:hofC’ positive recombinants.

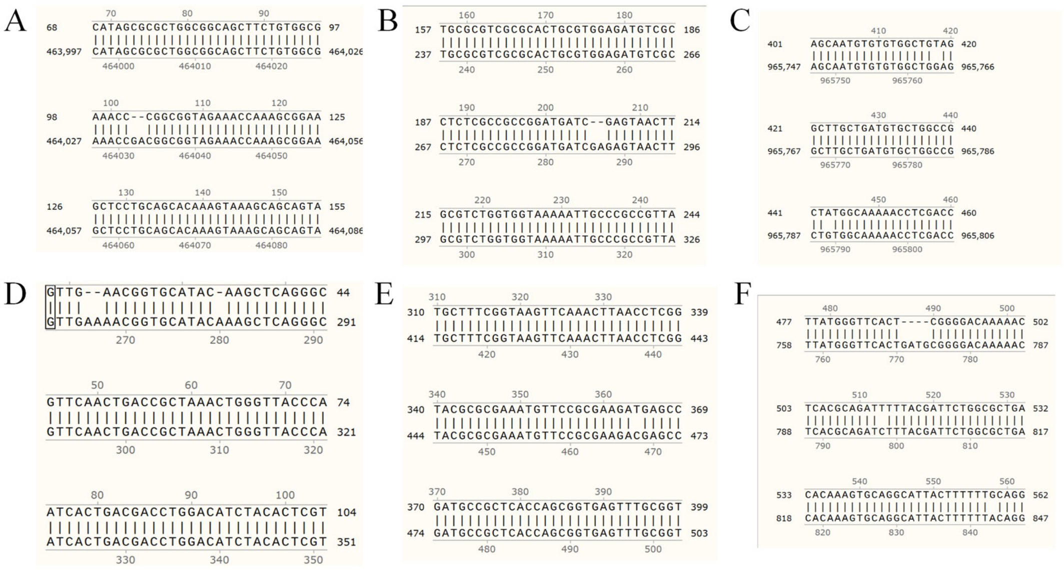

Figure S7. Sequence alignment maps of gene mutation sites

Note: A, B, C, D, E, F represented alignment diagram of sequencing results of E. coli DH5α:ybaV’, DH5α:rpoS’, DH5α:ycaI’, DH5α:ompA’, DH5α:ompR’, DH5α:hofC’ positive recombinants, correspondingly.

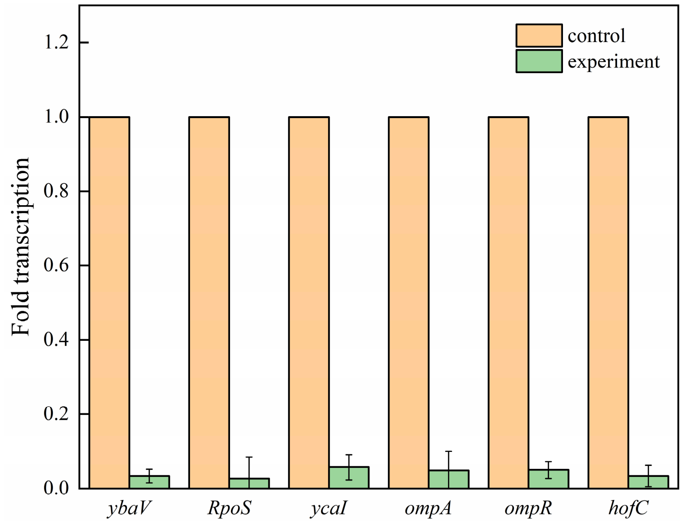

Figure S8. qPCR expression levels in gene knockout and control groups of cells
